# The Relative Merits of Posterior Surgical Treatments for Multi-Level Degenerative Cervical Myelopathy Remain Uncertain: Findings from a Systematic Review

**DOI:** 10.3390/jcm10163653

**Published:** 2021-08-18

**Authors:** Xiaoyu Yang, Aref-Ali Gharooni, Rana S. Dhillon, Edward Goacher, Edward W. Dyson, Oliver Mowforth, Alexandru Budu, Guy Wynne-Jones, Jibin Francis, Rikin Trivedi, Marcel Ivanov, Sashin Ahuja, Kia Rezajooi, Andreas K. Demetriades, David Choi, Antony H. Bateman, Nasir Quraishi, Vishal Kumar, Manjul Tripathi, Sandeep Mohindra, Erlick A. Pereira, Giles Critchley, Michael G. Fehlings, Peter J. A. Hutchinson, Benjamin M. Davies, Mark R. N. Kotter

**Affiliations:** 1 Division of Neurosurgery, Department of Clinical Neurosciences, University of Cambridge, Cambridge CB0 0GG, UK; xy393@outlook.com (X.Y.); aag56@cam.ac.uk (A.-A.G.); om283@cam.ac.uk (O.M.); Jibin.Francis@gmail.com (J.F.); RikinTrivedi@hotmail.com (R.T.); pjah2@cam.ac.uk (P.J.A.H.); mrk25@cam.ac.uk (M.R.N.K.); 2 Department of Neurosurgery, Leiden University Medical Centre, 2333ZA Leiden, The Netherlands; 3 Department of Neurosurgery, St Vincent’s Hospital Melbourne, Melbourne, VIC 3065, Australia; ranadhillon@email.com; 4 Department of Neurosurgery, Royal Hallamshire Hospital, Sheffield S10 2JF, UK; edward.goacher@doctors.org.uk; 5 Victor Horsley Department of Neurosurgery, National Hospital for Neurology & Neurosurgery, London WC1N 3BG, UK; edwarddyson@nhs.net; 6 Department of Neurosurgery, Sheffield Teaching Hospitals National Health Service Foundation Trust, Sheffield S10 2JF, UK; budu.alexandru@gmail.com (A.B.); marcel.ivanov@nhs.net (M.I.); 7 Department of Orthopaedics, The Newcastle upon Tyne Hospitals NHS Foundation Trust, Newcastle upon Tyne NE7 7DN, UK; GuyWynneJones@gmail.com; 8 Welsh Centre for Spinal Surgery & Trauma, University hospital of Wales, Cardiff CF14 4XW, UK; sashinahuja@gmail.com; 9 Spinal Surgery Unit, Royal National Orthopaedic Hospital, Stanmore HA7 4LP, UK; kia.rezajooi@rnoh.nhs.uk; 10 Edinburgh Spinal Surgery Outcome Studies Group, Department of Neurosurgery, Royal Infirmary of Edinburgh, Edinburgh EH16 4SA, UK; demetriades@gmail.com; 11 Department of Neurosurgery, National Hospital for Neurology and Neurosurgery, University College London Hospitals, London WC1N 3BG, UK; david.choi@nhs.net; 12 Royal Derby Spinal Centre, Royal Derby Hospital, Derby DE22 3NE, UK; abateman@doctors.org.uk; 13 Nottingham Centre for Spinal Studies and Surgery, Queens Medical Centre, Nottingham University Hospitals, Nottingham NG7 2UH, UK; Nasir.Quraishi@nuh.nhs.uk; 14 Department of Orthopaedics, Post Graduate Institute of Medical Education and Research (PGIMER), Chandigarh 160012, India; drkumarvishal@gmail.com; 15 Department of Neurosurgery, Post Graduate Institute of Medical Education and Research (PGIMER), Chandigarh 160012, India; drmanjultripathi@gmail.com (M.T.); sandeepneuro@gmail.com (S.M.); 16 Neurosciences Research Centre, Institute of Molecular and Clinical Sciences, St George’s, University of London, London WC1E 7HU, UK; erlick.pereira2@nhs.net; 17 Brighton and Sussex Medical School, South East Neurosurgery and South East Spinal Surgery, University Hospitals Sussex NHS Foundation Trust, Brighton BN1 9PX, UK; giles.critchley@nhs.net; 18 Division of Neurosurgery, Toronto Western Hospital, University Health Network, Toronto, ON M5T 2S8, Canada; Michael.Fehlings@uhn.ca; 19 Anne McLaren Laboratory for Regenerative Medicine, Welcome Trust MRC Cambridge Stem Cell Institute, University of Cambridge, Cambridge CB2 0PY, UK

**Keywords:** cervical spine, multi-level, myelopathy, laminoplasty, laminectomy, fusion, degenerative cervical myelopathy

## Abstract

Objectives: To assess the reporting of study design and characteristics in multi-level degenerative cervical myelopathy (DCM) treated by posterior surgical approaches, and perform a comparison of clinical and radiographic outcomes between different approaches. Methods: A literature search was performed in Embase and MEDLINE between 1995–2019 using a sensitive search string combination. Studies were selected by predefined selection criteria: Full text articles in English, with >10 patients (prospective) or >50 patients (retrospective), reporting outcomes of multi-level DCM treated by posterior surgical approach. Results: A total of 75 studies involving 19,510 patients, conducted worldwide, were identified. Laminoplasty was described in 56 studies (75%), followed by laminectomy with (36%) and without fusion (16%). The majority of studies were conducted in Asia (84%), in the period of 2016–2019 (51%), of which laminoplasty was studied predominantly. Twelve (16%) prospective studies and 63 (84%) retrospective studies were identified. The vast majority of studies were conducted in a single centre (95%) with clear inclusion/exclusion criteria and explicit cause of DCM. Eleven studies (15%) included patients with ossification of the posterior longitudinal ligament exclusively with cohorts of 57 to 252. The clinical and radiographic outcomes were reported with heterogeneity when comparing laminoplasty, laminectomy with and without fusion. Conclusions: Heterogeneity in the reporting of study and sample characteristics exists, as well as in clinical and radiographic outcomes, with a paucity of studies with a higher level of evidence. Future studies are needed to elucidate the clinical effectiveness of posterior surgical treatments.

## 1. Introduction

Degenerative cervical myelopathy (DCM) is a common and disabling condition, caused by arthritic changes in the cervical spine that compress and injure the cervical spinal cord. This results in functional impairment of the spinal cord that progresses at various rates and patterns, most commonly in a stepwise deterioration with periods of stable symptoms [1]. DCM is estimated to affect up to 2.3% [2] of adults and leads to progressive loss of dexterity, gait disturbance, imbalance, bladder disturbance, and occasionally incontinence and tetraplegia [1]. Surgery is currently the only treatment shown to alter the natural history of the disease: removing the mechanical compression on the spinal cord can stop disease progression and typically offer meaningful, albeit incomplete, recovery. There are a number of different surgical approaches and techniques in use. International guidelines currently recommend surgery for moderate (mJOA 12–14) to severe impairment (mJOA ≤ 11) and any progressive disease [3].

These guidelines leave the choice of procedure at the discretion of the operating surgeon, which reflects an uncertainty within scientific evidence over the relative merits and contra/indications for specific procedures [4]. Understanding these nuances is a recognised research priority by AO Spine RECODE DCM (aospine.org/recode) [5]; ‘Individualising Surgery’, and the need to address specific sub-questions of surgery, for example as is being evaluated in cervical spondylotic myelopathy (CSM) surgery, a randomised controlled trial (RCT) of anterior versus posterior surgery [6].

A further area of uncertainty remains the role of stabilisation or reconstruction after decompression. For DCM treated posteriorly, the typically used techniques are laminectomy, laminoplasty or laminectomy and fusion [7]. These techniques all provide posterior decompression but have differing approaches to stabilisation: laminectomy includes no stabilisation [8], laminoplasty (with several variations) uses a construct to float and retain the dorsal elements posteriorly [9] whilst laminectomy and fusion uses instrumentation to rigidly stabilise the spinal column [10,11,12,13,14]. These techniques therefore represent contrasting views on the contribution of dynamic instability to the pathogenesis of DCM, and the significance and role of retaining range of motion (ROM) versus preventing secondary cervical deformity.

Whether or not this is significant to patients is uncertain [10,15], with conflicting evidence [16,17,18,19] and recommendations [8,14,20,21], leading to widespread variation in clinical practice [22,23]. Although widely used, there has been no prospectively powered comparison of these techniques [4]. Furthermore, much of the evidence comes from cohorts including single level disease [24,25]. One assumption is the inherent biomechanical implications for posterior surgery are magnified when treating multiple levels, and this is most likely where any divergence would be most significant. This subgroup of multi-level DCM is therefore underrepresented in DCM literature [24,25] and represents an important knowledge gap in particular given the popularity of a global preference for posterior techniques for multi-level DCM [7].

The objectives of this study were therefore to describe the current evidence for posterior surgical treatment of multi-level DCM in terms of the range of outcome measures and the manner in which they were reported to inform the design of a prospective trial. Furthermore, where possible, to compare the clinical and radiographic outcomes between different posterior approaches.

## 2. Materials and Methods

The systematic review was conducted in accordance with the Preferred Reporting Items for Systematic Reviews and Meta-Analyses: the PRISMA Statement [26]. Due to heterogenous outcome reporting, a formal meta-analysis was not possible in this study and comparisons were made descriptively [27].

### 2.1. Literature Search and Selection

Up to 11th November 2019, the electronic databases Embase [Ovid] and MEDLINE [Ovid] were searched using the search strategies as shown in Table S1. Two of the authors (XY and AG) independently evaluated the articles by title, abstract, or full article, where necessary, to select the studies that met the predefined selection criteria. Selection criteria were stated as follows:

- Prospective study with more than 10 patients or retrospective study with more than 50 patients;
- Including multi-level DCM, defined as 2 or more levels;
- Including posterior surgical treatment;
- English, full text;
- Articles published since 1st January 1995.

Animal studies, letters and editorials were excluded from this study. Reference screening and citation tracking were performed on the identified articles and as a final check, the reviews found in the search were studied to make sure no relevant articles were missed. Any discrepancy in selection between the two reviewers was resolved by a third reviewer (BD). Descriptive statistics were used to report frequency and proportion of outcome measures. Statistical comparisons were made using the Chi-Squared test, with significance set at *p* = 0.05.

### 2.2. Data Extraction

Data extraction was performed by two independent reviewers (XY and AG), using a piloted extraction template covering study characteristics, design, participant characteristics, clinical outcome and radiographic outcome. Extracted data underwent a narrative synthesis and was presented with summary tables.

### 2.3. Quality Assessment

Two reviewers (XY and AG) independently appraised each publication according to study design. None of the studies found in our review of the literature were randomised trials. Nonrandomised observational studies were evaluated utilising the New Castle Ottawa Scale to evaluate the validity of each. Discrepancies between the two reviewers were addressed by a joint re-evaluation of the original article.

## 3. Results

### 3.1. Characteristics of Studies

**Figure 1:**
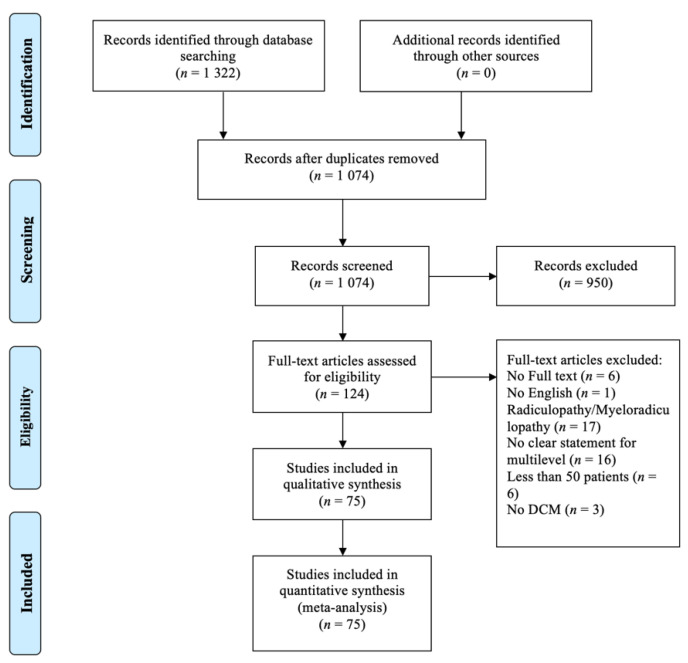
PRISMA flow diagram of search strategy.

Of the 1322 articles identified, 1074 original articles were left after removing duplicates. Following abstract and title review, 124 articles were shortlisted. After reviewing the full text, 75 articles were included in this study, assessing 19,510 patients (Figure 1). Of the 75 included articles, 18 studies reported the comparison between anterior and posterior approach, of which the data regarding posterior approach was extracted and included in this review.

**Figure 2:**
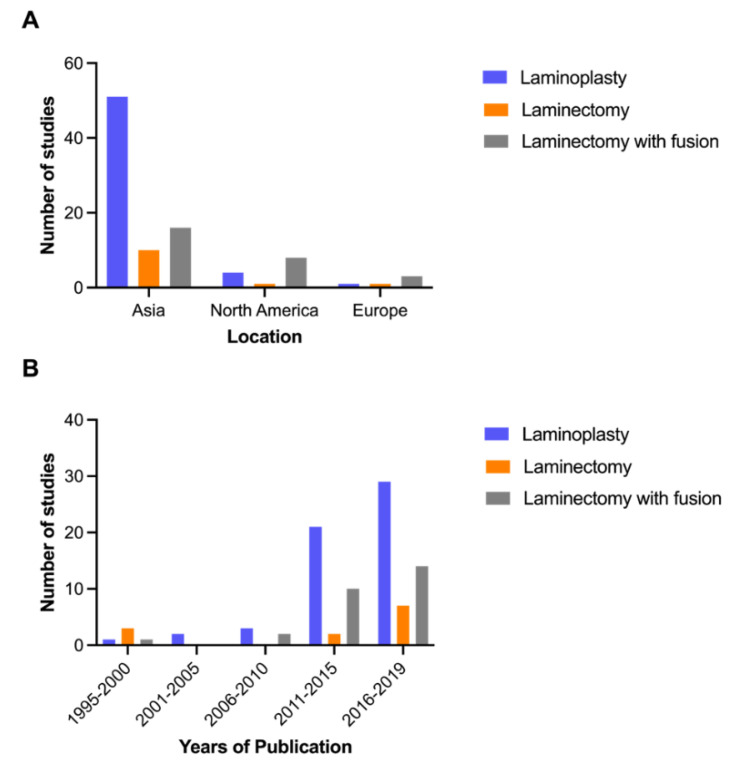
Location (**A**) and trend (**B**) of published research.

Laminoplasty was described in 56 studies (75%), whereas laminectomy with fusion in 27 (36%), and laminectomy without fusion in 12 (16%). The majority of studies were conducted in Asia (*n* = 63, 84%), followed by North America (*n* = 8, 11%) and Europe (*n* = 4, 5%) (Figure 2A). The articles were mainly published in the period of 2016–2019 (*n* = 38, 51%) and 2011–2015 (*n* = 26, 35%), of which laminoplasty was studied predominantly (Figure 2B). The sample size ranged from 51 to 1025.

### 3.2. Data Quality

The New Castle Ottawa Scale was used to assess the quality of each study due to its high content validity and inter-rater reliability. One study was allotted three stars, three studies four stars, five studies five stars, and two studies were assessed and awarded six stars (Table 1).

### 3.3. Study Design, Patient Selection and Reporting Differences

Twelve (16%) studies were conducted prospectively, and 63 (84%) retrospective studies were identified. The vast majority of studies were conducted in a single centre (*n* = 71, 95%), three were in multiple centres, and the design of the other study is unknown. Of the 75 studies, 45 (60%) documented that ethical approval was obtained, including one study which held the waiver for ethical approval. Clear inclusion and exclusion criteria were defined in 72 (96%) and 59 (79%) studies, respectively. All of the included studies described the cause of cervical myelopathy, of which 11 (15%) studies included patients suffering ossification of the posterior longitudinal ligament (OPLL) exclusively with sample sizes of 57 to 252, while other studies comprised patients with DCM. The number of levels involved in the diagnosis of multi-DCM was specified in 36 studies describing it as two or more than two levels (*n* = 3, 4%), three or more than three levels (*n* = 28, 37%), four levels (*n* = 4, 5%) and five levels (*n* = 1, 1%).

Reporting differences were noted when comparing prospective with retrospective studies (Tables S2–S4). When compared to retrospective studies, prospective studies were more likely to report the duration of symptom (*p* = 0.047) and the result of dynamic X-rays (*p* = 0.023).

### 3.4. Comparison between Laminoplasty and Laminectomy with Fusion

Six retrospective studies [28,29,30,31,32,33] compared laminoplasty to laminectomy with fusion with sample sizes ranging from 56 to 141 patients (Table 2). The surgical treatment was decided based on (1) surgeon’s choice: Highsmith et al. [30] chose patients with more facet pathology to undergo laminectomy and fusion, while Yang et al. [33] preferred patients with large anterior osteophytes, facet degeneration, and the continuous type of OPLL to receive laminectomy and fusion; (2) radiographic parameters: Ha et al. [29] preferred laminectomy with fusion for patients with straight or lordotic cervical curvature and segmental instability, and those with severe cord compression caused by OPLL, while Stephens et al. [32] preferred those who demonstrated any amount of C2–7 kyphosis to undergo laminectomy and fusion; or (3) the combination of both (further details were not available) [31]. Of the six studies, two [29,31] included patients with exclusively OPLL, while others comprised patients with DCM.

Ajiboye et al. [28] reported that there was no difference observed in modified Japanese orthopaedic association (mJOA) score between two groups, while laminectomy with fusion was associated with larger interval regression in disc-osteophyte complex size measured on magnetic resonance imaging (MRI) compared to laminoplasty. Ha et al. [29] observed similar improvements in Health-related quality of life (HRQOL), JOA recovery, and visual analog scale (VAS) in both groups, whilst neck disability index (NDI) improved more significantly in the laminoplasty group. Laminoplasty preserved cervical lordosis, ROM and C2–C7 sagittal vertical axis (SVA) more than laminectomy with fusion group, but the progression of OPLL was suppressed by stabilization using instrumented fusion. Highsmith et al. [30] reported comparable improvements in Nurick scores, mJOA, and Odom outcomes, and comparable radiographic outcomes between groups. They also noted improved VAS neck pain in laminectomy with fusion, though at higher cost (3 times) and increased complications (2 times), compared to laminoplasty. Stephens et al. [32] found that overall pain scores and mJOA improved significantly in both groups. Improved NDI and the loss of lordosis were found in laminoplasty group. Yang et al. [33] reported that the neurological functional recovery (JOA and Nurick scores) was similar between groups. Neck function (NDI and VAS) was worse in the laminectomy and fusion group, although with the achievement of a greater extent of enlargement of the spinal canal and spinal cord drift, compared with laminoplasty. Lee et al. [31] did not report clinical outcome but demonstrated that laminectomy with fusion had the effect of reducing OPLL growth rate compared with motion-preserving laminoplasty.

### 3.5. Comparison between Laminoplasty and Laminectomy without Fusion

Three studies [34,35,36] were conducted retrospectively by comparing laminoplasty to laminectomy alone with the sample sizes ranging from of 67 to 330 (Table 3). The surgeon-based treatment was recorded in only one study (no further details provided) [35]. Two studies [34,35] included patients with CSM and the other [36] enrolled patients with OPLL only.

Chang et al. [34] demonstrated similar clinical outcomes (NDI, JOA and VAS neck pain) between groups. Although shorter operation time and less blood loss was observed in the laminectomy group, Cobb angle and ROM significantly decreased at 1-year follow-up. Li et al. [35] compared laminectomy to French-door and open-door laminoplasty, and demonstrated a significantly improved Nurick score and reduced postoperative ROM in all groups at 1-year follow-up. However, French-door laminoplasty showed a higher bone union rate with smaller increased spinal cord volume compared to the other two groups. Yoo et al. [36] found no difference between laminoplasty and laminectomy in 73 patients with OPLL, neither on clinical outcomes (NDI and JOA), nor on radiographic outcomes (C2–7 Cobb angle, SVA, and T1 slope).

### 3.6. Comparison between Laminoplasty, Laminectomy with and without Fusion

Two retrospective studies reported comparison between laminoplasty, laminectomy with and without fusion, but none of them mentioned the allocation method (Table 4). Du et al. [37] studied 98 patients and reported that an excellent neurological improvement (JOA recovery rates ≥ 75 %) was achieved in patients with laminoplasty and laminectomy with fusion at 7 to 12 years follow-up, whilst a high incidence of axial symptoms (NDI) was found in the laminoplasty and laminectomy alone groups caused by loss of curvature index. In the fusion group, lateral mass screw fixation was demonstrated to effectively prevent loss of postoperative cervical curvature and therefore to reduce the incidence of axial symptoms. Lee et al. [38] investigated sagittal alignment and clinical outcome in 57 patients with CSM and OPLL, and found that cervical lordosis, C2–C7 Cobb angle, and cervical curvature index decreased gradually in all patients at minimum 2-year follow-up, with the exception of SVA which was maintained in laminectomy with fusion group. Clinical outcomes, NDI and VAS, improved in all patients. Neck pain was found to increase in laminoplasty in patients showing SVA more than 40 mm at baseline, and the progression of OPLL was observed more frequently in the laminectomy alone group than the group with fusion.

## 4. Discussion

### 4.1. Summary of Findings

DCM is a common cause of spinal cord injury, and many patients with DCM go on to develop progressive disease leading to neurological deficits and reduced quality of life.

This study has identified significant heterogeneity in the conduct and reporting of clinical research evaluating posterior surgery for multi-level DCM. This included variation in study design characteristics, such as the reporting of ethics committee approval, clear inclusion/exclusion criteria and population characteristics, such as the definition of multi-level and subtype of DCM. Most studies were conducted in Asia during recent years focusing on laminoplasty. Few studies made direct comparisons of techniques, and no high level of evidence, such as a RCT, was found. Due to the heterogeneous reporting of outcomes, it was a challenge to interpret these results and taken together this confirmed an important knowledge gap for surgeons.

### 4.2. Comparison between Posterior Approaches

As the most popular posterior surgical approach described in the literature, laminoplasty was compared to laminectomy with and without fusion. When compared to laminectomy with fusion, with various measurements evaluated, the clinical findings were heterogeneous and contradictory. However, two studies [29,31] reported the superiority of laminectomy with instrumented fusion at suppressing the progression of OPLL when compared with other procedures. One possible explanation is that the decrease in pulsations of the thecal sac and venous plexus after posterior fusion lead to the reduction in thickness of OPLL [39,40]. Another possibility is the removal of mechanical stimulus for cervical OPLL after posterior fusion possibly suppresses the progression of OPLL [41]. More research is still needed to draw a firm conclusion on this topic. When compared to laminectomy alone, although reported with various measurements, comparable clinical outcomes were demonstrated between groups. Cervical laminoplasty was introduced in Japan in the 1970s, with proposed advantages of protecting the spinal cord and preventing neurological deterioration by preservation of the posterior elements and stability [42,43]. However, this is still a controversial issue. In this systematic review it was not possible to show superior clinical outcomes for any particular posterior surgical procedure used to treat DCM as was the case in previous systematic reviews [44,45]. Although Du et al. [37] demonstrated laminectomy with fusion to have a JOA improvement with less incidence of axial symptoms in the comparison of three surgical approaches, this was not confirmed by Lee et al. [38]. Furthermore, a recent meta-analysis disputes this finding, which concluded that laminoplasty had fewer complications, a lower incidence of C5 palsy, better NDI scores and recovery outcomes compared to laminectomy with fusion [46]. Again, due to limited and heterogenous outcomes, no firm conclusion could be made.

Whilst the evidence base has largely focused on laminoplasty, especially in Asia, it is of note in clinical practice that the use of instrumented fusion has increased significantly. This is acknowledged by Deyo et al., who describe how the adoption of technology within spinal surgery has outstripped its rigorous evaluation [47]. More broadly, this is a recognised problem throughout surgery and underpins the IDEAL framework, and specifically the need to match innovation with evaluation [48].

Of note, CSM-S, a RCT of ventral versus dorsal surgery for DCM has recently reported [49]. In this trial, which randomised patients undergoing surgery for multi-level CSM (i.e., excluding OPLL) in the absence of kyphosis to an anterior or posterior approach in whom there was surgical equipoise, a planned subgroup analysis of laminoplasty (*n* = 28) vs. laminectomy and fusion (*n* = 69) occurred. The decision to perform a laminoplasty versus a laminectomy and fusion was at the surgeon’s discretion. In this subgroup, posterior instrumented fusion was associated with significantly higher adverse events (fusion, 29.0% [95% CI, 18.7%–41.2%]; laminoplasty, 10.7% [95% CI, 2.3%–28.2%]), increased opioid use (fusion, 65.2% [95% CI, 52.8%–76.3%]; laminoplasty, 39.3% [95% CI, 21.5%–59.4%]), and worse physical function at 2 years (estimated mean 5.8; 95% CI, 1.5–10.1; *p* = 0.01). This difference is greater than their defined MCID. Furthermore, the rate of recovery from instrumented fusion was slower, the short-term neck disability greater and return to work delayed. In fact, these outcomes amongst the laminoplasty subgroup broadly matched anterior surgical results.

### 4.3. Designing a Future Comparative Study

The results of this systematic review indicate that there is no high level of evidence to guide surgeons when considering a posterior surgical approach for patients with multi-level DCM. Although improved outcomes have been reported in laminectomy with fusion, considering the significant costs, additional skill, increased operative time and reduced ROM after surgery, its superior cost-effectiveness compared to laminectomy requires evaluation. Thus, a comparative study is needed to answer this question.

Although, no firm conclusion can be drawn in this review concerning the clinical effectiveness of posterior surgical treatments, it provides useful information which will facilitate the setting up of future comparative studies. All of three posterior approaches were effective when performed for patients with multilevel DCM. However, the indications of each approach were inconsistent, and some were even contradictory [29,32], paving the way for a randomised controlled trial. The majority of previous studies have a follow-up duration within 24 months, which seems to be pragmatic. Furthermore, various outcome measures have been used in previous studies, including clinical (neurological function assessment and neck pain score) and radiological alignment (X-rays).

Ideally, a three-armed RCT would examine the effectiveness of these posterior surgical treatments. Nevertheless, some existing disputes make it difficult to conduct, such as whether a Bonferroni or similar correction factor should be employed to decrease the likelihood of a type I error in the three-armed RCT. Additionally, given that all procedures are effective to some extent and the relative differences to be detected are likely to be small, this would significantly inflate the sample size. Thus, the initial step, to examine the fundamental question of whether or not stabilisation is required after posterior decompression may be an RCT of the two extremes: laminectomy alone versus laminectomy with fusion. Such a trial has been commissioned by the National Institute for Health Research within the UK, in part owing to a very limited use of laminoplasty in UK spinal practice: The POLYFIX-DCM trial (Posterior LaminectomY and FIXation for DCM) aims to offer the first fully powered, randomised evaluation of this question and will commence recruitment in January 2022. International sites and collaborators are sought.

### 4.4. Limitations

Due to various definitions of ‘multi-level DCM’, patients who received short-range decompression may have compared to those who underwent long-range surgeries in this review. However, it is still not clear whether there is a clinical significance between them. Besides, findings in this review were generalised from studies with CSM and OPLL, which are two different pathogenic factors for DCM. Due to the paucity of comparative data, further subgroup analysis was not possible. Furthermore, the follow-up of included studies may be inadequate (mostly 1–2 years), since adjacent segment disease and bony remodelling may take years to occur and is arguably the most important difference between fusion and non-fusion surgery. This study was designed to focus on contemporary and large sample studies, and those articles with non-English language were excluded. The global representation of included studies suggests that the foreign language exclusion is unlikely to be significant. Indeed, the authors propose that assessment of 25 years of published data of large sample studies, is representative of current practice.

## 5. Conclusions

Studies evaluating posterior surgery for multi-level DCM demonstrate heterogeneity in the reporting of definitions, sample characteristics, as well as in clinical and radiographic outcomes. To date, no studies with a high level of evidence exist. This represents an important knowledge gap, supporting an individualised approach to DCM surgery, and a current leading research priority as identified by AO Spine RECODE DCM (aospine.org/recode).

## Figures and Tables

**Table 1:** Study Quality.

Article	Years of Publication	Selection				Comparability	Outcome			Total Score
		Exposed Cohort	Non-Exposed Cohort	Ascertainment of Exposure	Outcome of Interest		Assessment of Outcome	Length of Follow-Up	Adequacy of Follow-Up	
Ajiboye et al.	2017	-	-	*	-	*	-	-	-	3 *
Chang et al.	2017	-	-	*	-	*	-	*	*	5 *
Du et al.	2013	-	-	*	-	*	*	*	*	5 *
Ha et al.	2019	*	*	*	-	*	-	*	*	6 *
Highsmith et al.	2011	-	-	*	-	*	-	*	*	4 *
Lee et al.	2016	-	-	*	-	*	-	*	*	4 *
Lee et al.	2018	-	-	*	-	*	-	*	*	4 *
Li et al.	2019	-	-	*	-	*	-	*	*	5 *
Stephens et al.	2017	*	*	*	-	*	-	*	*	5 *
Yang et al.	2013	-	-	*	-	**	*	*	*	6 *
Yoo et al.	2017	-	-	*	-	**	-	*	*	5 *

**Table 2:** Studies describing laminoplasty versus laminectomy with fusion.

Article	Years of Publication	Sample Size	Study Design	Allocation Basis	Cause of Myelopathy	Function Outcome	Time Point	Radiographic Outcome	Time Point
Ajiboye et al.	2007	70	Retro	NA	CSM	mJOA	Pre- and post- operation	Disc-osteophyte complex size	Baseline and 10 months
								Cobb angle	Pre-operation
Ha et al.	2019	91	Retro	Radiological factors and age	OPLL	NDI JOA	Pre- and 2 years post-operation	C2–7 SVA C0–2 Cobb angle C2–7 Cobb angle T1 slope Total ROM	Pre-operation and 6,12,24 months
								Occupying ratio of the spinal canal	Pre-operation
								The thickness of the OPLL Progression of the thickness of the OPLL	Pre-operation and 24 months
								Signal intensity changes	Pre-operation
Highsmith et al.	2011	56	Retro	Surgeon-based	Cervical stenotic myelopathy	mJOA Nurick Odom	42 months	Cervical lordosis ROM	Pre-operation and 42 months
Lee et al.	2018	83	Retro	Cervical lordosis Neck pain Surgeon’s preference	OPLL	NA	-	Cervical curvature ROM	Pre-operation and 2 years
								OPLL volume	Pre-operation
Stephens et al.	2017	137	Retro	Neck pain C2–7 sagittal angle	Cervical myelopathy	NDI mJOA	Pre-operation 6 weeks, 6 and 12 months	C2–7 Cobb angle T1 slope C2–7 SVA Forward pitch Axial canal diameter Miyazaki Spondylosis score	Pre-operation and 12 months
Yang et al.	2013	141	Retro	Surgeon-based	Cervical stenotic myelopathy	mJOA Nurick NDI	Pre- and 24 months	ROM Cervical curvature	Pre- and 24 months
								Osseous fusion	6 months postoperatively
								Area of dural sac, increase in area, spinal cord drift	Pre-operation, 6 and 24 months

NA: Not applicable; CSM: Cervical spondylotic myelopathy; mJOA: Modified Japanese orthopaedic association score; OPLL: Ossification of the posterior longitudinal ligament; NDI: Neck disability index; JOA: Japanese orthopaedic association score; SVA: Sagittal vertical axis; ROM: Range of motion.

**Table 3:** Studies describing laminoplasty versus laminectomy alone.

Article	Years of Publication	Sample Size	Study Design	Allocation Basis	Cause of Myelopathy	Function Outcome	Time Point	Radiographic Outcome	Time Point
Chang et al.	2017	67	Retro	NA	CSM	JOA NDI	Pre-operation, and 12 months	C2–7 Cobb angle ROM	Pre-operation, post-operation, and 12 months
								Maximal cord compression	Pre-operation
								Spinal canal expansion	Pre-operation and 6 weeks
Li et al.	2019	330	Retro	Surgeon-based	CSM	Nurick	12 months	Cervical lordosis ROM	Pre-operation and 12 months
								Bony fusion	6 and 12 months
								Spinal cord volume	1 week
Yoo et al.	2017	73	Retro	NA	OPLL	JOA NDI	Pre-operation and >2 years	C2–7 Cobb angle SVA T1 slope	Pre-operation and >2 years

NA: Not applicable; CSM: Cervical spondylotic myelopathy; JOA: Japanese orthopaedic association score; NDI: Neck disability index; ROM: Range of motion; OPLL: Ossification of the posterior longitudinal ligament; SVA: Sagittal vertical axis.

**Table 4:** Studies describing laminoplasty versus laminectomy with and without fusion.

Article	Years of Publication	Sample Size	Study Design	Allocation Basis	Cause of Myelopathy	Function Outcome	Time Point	Radiographic Outcome	Time Point
Du et al.	2013	98	Retro	NA	DCM and OPLL	JOA NDI	Pre-operation and 7 to 12 years	Curvature index	Pre-operation and 7 to 12 years
Lee et al.	2016	57	Retro	NA	CSM and OPLL	NDI	Pre-operation and 2 years	Curvature index C2–C7 SVA C2–C7 Cobb angle	Pre-operation and 2 years

NA: Not applicable; DCM: Degenerative cervical myelopathy; OPLL: Ossification of the posterior longitudinal ligament; JOA: Japanese orthopaedic association score; NDI: Neck disability index; CSM: Cervical spondylotic myelopathy; ROM: Range of motion; SVA: Sagittal vertical axis.

## Data Availability

Not applicable.

## Supplementary Materials

The following are available online at https://www.mdpi.com/article/10.3390/jcm10163653/s1, Table S1 Search strategy; Table S2 Differences in reporting characteristics and study design; Table S3 Differences in reporting clinical outcomes; Table S4 Differences in reporting radiographic outcomes.
