# Supplementary material for: The Relative Merits of Posterior Surgical Treatments for Multi-Level Degenerative Cervical Myelopathy Remain Uncertain: Findings from a Systematic Review
Source: J Clin Med. 2021 Aug 18;10(16):3653. doi: 10.3390/jcm10163653 (PMC8397218; doi:10.3390/jcm10163653)
Supplement: Supplementary file 1 [file jcm-10-03653-s001.zip › jcm-1306648-supplementary.pdf]

Journal of
Clinical Medicine
MDPI

Table S1 Search strategy

Medline

1 exp Cervical Vertebrae/ or exp Cervical Cord/ or cervical.mp. or (phrenic nucleus or accessory nucleus).mp. or (("Japanese Orthop?edic Association" adj2 score*) or (joa adj2 score*)).mp. (254878)

2 myelopath*.mp. or exp Spinal Cord Diseases/ or (spinal cord adj3 (diseas* or disorder*)).mp. or myeloradiculopath*.mp. or spondylomyelopath*.mp. or spondylomyeloradiculopath*.mp. or (Spinal Cord adj3 Compress*).mp. or exp Spinal Cord Compression/ (138462)

3 1 and 2 (18807)

4 exp "Ossification of Posterior Longitudinal Ligament"/ (909)

5 3 or 4 (19257)

6 exp Atlanto-Occipital Joint/ or exp Arteriovenous Fistula/ or exp Radiotherapy/ or exp Vitamin B 12/ or exp Radiation/ or exp Radiation Injuries/ or exp Re-Irradiation/ or exp Craniospinal Irradiation/ or exp Whole-Body Irradiation/ or exp Motor Neuron Disease/ or exp Amyotrophic Lateral Sclerosis/ or exp Neoplasm Metastasis/ or exp Hemangioma/ or exp neoplasm/ or exp metastasis/ or exp Nervous System Malformations/ or exp "autoimmune diseases of the nervous system"/ or exp "congenital, hereditary, and neonatal diseases and abnormalities"/ or exp virus diseases/ (5622283)

7 5 not 6 (14168)

8 (Multi-level* or multilevel* or 2-level* or 3-level* or 4-level* or 5-level* or three-level* or four-level* or five-level* or ((two or three or four or five or multi* or "2" or "3" or "4" or "5" or all or alternate or each) adj3 level*)).mp. [mp=title, abstract, original title, name of substance word, subject heading word, floating sub-heading word, keyword heading word, organism supplementary concept word, protocol supplementary concept word, rare disease supplementary concept word, unique identifier, synonyms] (410842)

9 exp Laminoplasty/ or exp laminectomy/ or (Posterior* or laminectom* or laminotom* or laminoplast* or laminaplast*).mp. (300782)

10 7 and 8 and 9 (885)

Embase

1 exp *cervical vertebra/ (725)

2 exp *cervical spinal cord/ (1939)

3 cervical.ti,ab. (271463)

4 (phrenic nucleus or accessory nucleus).ti,ab. (252)

5 (("Japanese Orthop?edic Association" adj2 score*) or (joa adj2 score*)).ti,ab. (3118)

6 1 or 2 or 3 or 4 or 5 (273340)

7 myelopath*.ti,ab. (16741)

8 exp *spinal cord disease/ (152842)

9 (spinal cord adj3 (diseas* or disorder*)).ti,ab. (3097)

10 (myeloradiculopath* or spondylomyelopath*).mp. or spondylomyeloradiculopath*.ti,ab. [mp=title, abstract, heading word, drug trade name, original title, device manufacturer, drug manufacturer, device trade name, keyword, floating subheading word, candidate term word] (831)

11 (Spinal Cord adj3 Compress*).ti,ab. (9525)

12 exp *spinal cord compression/ (5585)

13 7 or 8 or 9 or 10 or 11 or 12 (166999)

14 6 and 13 (20321)

15 exp *ligament calcinosis/ (1051)

16 (Ossification adj2 "Posterior Longitudinal Ligament*").ti,ab. (376)

17 15 or 16 (1252)

18 14 or 17 (21108)

19 exp *atlantooccipital joint/ (999)

20 exp *arteriovenous fistula/ (15811)

21 exp *radiotherapy/ (201305)

22 exp *cyanocobalamin/ (12005)

23 exp *radiation/ (207193)

24 exp *radiation injury/ (31268)

25 exp *re-irradiation/ (605)

26 exp *craniospinal irradiation/ (341)

27 exp *whole body radiation/ (6668)

28 exp *motor neuron disease/ (25204)

29 exp *amyotrophic lateral sclerosis/ (20987)

30 exp *metastasis/ (203702)

31 exp *hemangioma/ (24775)

32 exp *malignant neoplasm/ (2400042)

33 exp *nervous system malformation/ (52132)

34 exp *neurologic disease/ (2071985)

35 exp *"genetic and familial disorders"/ (674205)

36 exp *virus infection/ (701613)

37 19 or 20 or 21 or 22 or 23 or 24 or 25 or 26 or 27 or 28 or 29 or 30 or 31 or 32 or 33 or 34 or 35 or 36 (5748333)

38 18 not 37 (4369)

39 (Multi-level* or multilevel* or 2-level* or 3-level* or 4-level* or 5-level* or three-level* or four-level* or five-level* or ((two or three or four or five or multi* or "2" or "3" or "4" or "5" or all or alternate or each) adj3 level*)).ti,ab. (540443)

40 (Posterior* or laminectom* or laminotom* or laminoplast* or laminaplast*).ti,ab. (363188)

41 exp *laminoplasty/ (1002)

42 exp *laminectomy/ (3417)

43 40 or 41 or 42 (364352)

44 38 and 39 and 43 (437)

Table S2 Differences in reporting characteristics and study design

	Prospective	Retrospective	p	Total
Ethics	10 (83.3%)	35 (55.6%)	0.108#	45 (60%)
Multicentre	1 (8.3%)	2 (3.2%)	0.417#	3 (4.1%)
Inclusion criteria	12 (100%)	60 (95.2%)	1.0#	72 (96%)
Exclusion criteria	11 (91.7%)	48 (76.2%)	0.442#	59 (78.7%)
Cause of myelopathy	12 (100%)	63 (100%)	-	75 (100%)
Comorbidity mentioned	5 (41.7%)	11 (17.5%)	0.116#	16 (21.3%)
Symptom duration	8 (66.7%)	20 (31.7%)	0.047*	28 (37.3%)
Disease level(s)	11 (91.7%)	51 (81%)	0.679#	62 (82.7%)
Number of treated levels	9 (75%)	45 (71.4%)	1.0#	54 (72%)
Age	11 (91.7%)	60 (95.2%)	0.510#	71 (94.7%)
Sex	12 (100%)	57 (90.5%)	0.581#	69 (92%)
Operation time	8 (66.7%)	31 (49.2%)	0.267	39 (52%)
LOS	1 (8.3%)	17 (27%)	0.273#	18 (24%)
Blood loss	9 (75%)	32 (50.8%)	0.123#	41 (54.7%)
Previous surgery reported	9 (75%)	30 (47.6%)	0.082#	39 (52%)
Complication reported	7 (58.3%)	47 (74.6%)	0.299#	54 (72%)
Mortality reported	2 (16.7%)	6 (9.5%)	0.606#	8 (10.7%)
Reoperation/revision	3 (25%)	24 (38.1%)	0.519#	27 (36%)

#: Fisher's Exact Test

*: <0.05

LOS: Length of stay

Table S3 Differences in reporting clinical outcomes

	Prospective	Retrospective	p	Total
Clinical outcome assessed§	11 (91.7%)	60 (95.2%)	0.510#	68 (90.7%)
QOL assessed	1 (8.3%)	5 (7.9%)	1.0#	8 (8.0%)
Function assessed	11 (91.7%)	57 (90.5%)	1.0#	68 (90.7%)
Multiple function assessed	1 (8.3%)	18 (28.6%)	0.275#	19 (25.3%)
SF-36 assessed	0	3 (4.8%)	1.0#	3 (4.0%)
SF-36 reported	0	3 (4.8%)	1.0#	3 (4.0%)
JOA assessed	10 (83.3%)	47 (74.6%)	0.719#	57 (76%)
JOA reported	10 (83.3%)	46 (73%)	0.719#	56 (74.7%)
JOA recovery rate used	9 (75%)	35 (55.6%)	0.338#	44 (58.7%)
mJOA assessed	1 (8.3%)	7 (11.1%)	1.0#	8 (10.7%)
mJOA reported	1 (8.3%)	6 (9.5%)	1.0#	7 (9.3%)
Nurick assessed	0	12 (19%)	0.195#	12 (16%)
Nurick reported	0	12 (19%)	0.195#	12 (16%)
NDI assessed	1 (8.3%)	15 (23.8%)	0.442#	16 (21.3%)
NDI reported	1 (8.3%)	15 (23.8%)	0.442#	16 (21.3%)
Pain score	1 (8.3%)	19 (30.2%)	0.164#	20 (26.7%)
NRS	1 (8.3%)	1 (1.6%)	0.296#	2 (2.7%)
VAS	1 (8.3%)	24 (38.1%)	0.052#	25 (33.3%)

§: Including either a symptom, functional outcome or pain score was assessed or reported

#: Fisher's exact test

*: <0.05

QOL: Quality of life

SF-36: 36-Item short form survey

JOA: Japanese orthopaedic association score

mJOA: Modified Japanese orthopaedic association score

NDI: Neck disability index

NRS: Numerical rating scales

VAS: Visual analog scale

Table S4 Differences in reporting radiographic outcomes

	Prospective	Retrospective	P	Total
Baseline image§	9 (75%)	52 (82.5%)	0.686#	61 (81.3%)
MRI assessed	9 (75%)	32 (50.8%)	0.205	41 (54.7%)
MRI reported	1 (8.3%)	15 (23.8%)	0.442#	16 (21.3%)
MRI criteria	0	2 (3.2%)	1.0#	2 (2.7%)
CT assessed	3 (25%)	22 (34.9%)	0.704#	25 (33.3%)
CT reported	0	8 (12.7%)	0.341#	8 (10.7%)
CT criteria	0	2 (3.2%)	1.0#	2 (2.7%)
X-ray assessed	9 (75%)	48 (76.2%)	1.0#	57 (76%)
X-ray reported	8 (66.7%)	45 (71.4%)	0.739#	53 (70.7%)
X-ray criteria	0	3 (4.8%)	1.0#	3 (4%)
Dynamic X-rays assessed	7 (58.3%)	26 (41.3%)	0.348	33 (44%)
Dynamic X-rays reported	7 (58.3%)	21 (33.3%)	0.116#	28 (37.3%)
Dynamic X-rays criteria	0	1 (1.6%)	1.0#	1 (1.3%)
Radiographic outcome§	10 (83.3%)	56 (88.9%)	0.630#	66 (88%)
MRI assessed	4 (33.3%)	27 (42.9%)	0.751#	31 (41.3%)
MRI reported	4 (33.3%)	20 (31.7%)	1.0#	24 (32%)
CT assessed	1 (8.3%)	22 (34.9%)	0.091#	23 (30.7%)
CT reported	1 (8.3%)	17 (27%)	0.273#	18 (24%)
X-ray assessed	10 (83.3%)	51 (81%)	1.0#	61 (81.3%)
X-ray reported	9 (75%)	46 (73%)	1.0#	55 (73.3%)
Dynamic X-rays assessed	8 (66.7%)	19 (30%)	0.023#*	27 (41.5%)
Dynamic X-rays reported	8 (66.7%)	19 (30%)	0.023#*	27 (41.5%)

§: Including either X-ray, CT or MRI was assessed or reported

#: Fisher's exact test
